# A Note on Fatty Acids Profile of Meat from Broiler Chickens Supplemented with Inorganic or Organic Selenium

**DOI:** 10.1155/2017/7613069

**Published:** 2017-01-17

**Authors:** Marta del Puerto, M. Cristina Cabrera, Ali Saadoun

**Affiliations:** ^1^Department of Animal Production & Pastures, Nutrition and Food Quality Laboratory, Faculty of Agronomy, University of the Republic (UDELAR), E. Garzón 809, Montevideo, Uruguay; ^2^Physiology & Nutrition, Faculty of Sciences, University of the Republic (UDELAR), Iguá 4225, Montevideo, Uruguay

## Abstract

This investigation evaluated, in broiler chickens* Pectoralis* and* Gastrocnemius* muscles, the effect of the dietary supplementation with sodium selenite (0.3 ppm) versus selenomethionine (0.3 ppm), on the fatty acids composition, lipids indices, and enzymes indexes for desaturase, elongase, and thioesterase. The selenium reduced, in both muscles, the content of atherogenic fatty acids, C14:0 and C16:0, while it increased the C18:1 level. On the other hand, selenium increased, in both muscles, the content of C18:3n3 and EPA, but not DPA and DHA. No selenium effect was detected for PUFA/SFA, n-6, n-3, n-6/n-3, and atherogenic and thrombogenic indices. As for the enzyme indexes, a selenium effect is only detected for thioesterase. Taken together, the results highlight the potential effect of dietary selenium, mainly selenomethionine, in the modulation of the composition of fatty acids in chicken meat, in particular, reducing the content of atherogenic fatty acids and increasing the health promoting n-3 PUFA.

## 1. Introduction

Being a nonruminant animal, chickens tend to use, without significant changes, the lipids and the fatty acids present in a diet in order to fulfil its physiological needs for growth and muscle development [1]. When the chicken diet includes corn, soya meal, sunflower meal, and other green foods, it contributes to a relatively high content of fatty acids from the polyunsaturated fatty acids (PUFA) in the meat.

However, the presence of high levels of PUFA in chicken meat makes it more susceptible to the oxidation process and to undergoing alterations in smell, taste, and nutritional value. To counteract this concern, it is generally advised to add dietary antioxidants in chicken feed, to maintain the lipid stability in meat [2]. One of them, selenium, is a constituent of glutathione peroxidase, an essential enzyme in nutrients metabolism, and a first line of defense against the oxidation process [3]. In the feeding of farm animals, selenium is added in diet in inorganic as well as organic form. Until recently, the form of added selenium in farm animals diet had been inorganic, either selenite or selenate [4]. Nowadays, however, organic selenium attracts more interest as a supplement. This change could be linked to the fact that, apparently, organic selenium has a higher absorption rate than inorganic selenium in chickens [5].

It seems that selenium also has an interesting interaction with lipids metabolism. Indeed, Schäfer et al. [6] found that a selenium deficiency can interfere with the normal conversion of *α*-linolenic (ALA) into eicosapentaenoic (EPA) and docosahexaenoic (DHA) acids, which can result in an increased omega-6 : omega-3 ratio in the liver of rats. Furthermore, some studies showed that the supplementation with selenium in the diet of beef, pig, and chicken modified the fatty acid profiles of their meat [6–10]. Similar results were obtained for milk and colostrum [11]. This effect concerning selenium could be an interesting way of modifying the fatty acid profile of chicken meat, in addition to its protective effect against the oxidation process.

Therefore, the aim of this study is to determine the effect of the diet supplementation with selenium in inorganic form (sodium selenite, Se-Na), in comparison to the organic form (selenium methionine, Se-Met), on the fatty acids composition and some lipids indices (in terms of the health needs of consumers) of chicken meat from* Pectoralis* and* Gastrocnemius* muscles. Likewise, some enzyme indexes for desaturase, elongase, and thioesterase will be calculated in an attempt to detect any effect of the two kinds of selenium, on the lipids metabolism in the two muscles.

## 2. Materials and Methods

### 2.1. Animals and Diets

The animal care and handling were approved by the Committee on Experimental Animals of the Universidad de la República, Montevideo, Uruguay (CHEA), before the beginning of the experiment. Two hundred one-day-old male Ross birds were obtained from a commercial hatchery and reared until thirty-five days on a floor pen with wood shavings, in a climate-controlled room with a photoperiod of 23 hours of light/day. They were fed ad libitum with a commercial corn-soya diet (219 g·kg^−1^crude protein and 13.35 MJ·kg^−1^ of metabolizable energy). Tap water was given ad libitum. At thirty-five days, ninety birds were selected on homogenous weight basis and assigned randomly into three groups of thirty birds each. The birds were located in ten experimental pens (90 cm × 90 cm) with wood shavings as litter. Each pen located three birds, fed ad libitum, with one of the experimental diets, until slaughtering. A corn-soya based diet (Table 1) was formulated to meet the nutrient requirements for finishing male broilers [12] and was considered as the basal diet, not supplemented with selenium (Table 1). The other two diets were supplemented with selenium (Table 1) from an inorganic source (0.3 ppm Se, as sodium selenite, Se-Na in text and tables) or an organic source (0.3 ppm Se, as selenomethionine, Se-Met in text and tables). Dry matter, crude protein, fat, and fibre analysis of feed were carried out according to AOAC [13]. At fifty-six-days-old, all the birds were slaughtered at our own experimental abattoir. Preharvest handling, transportation (transportation time was 3 minutes), and slaughtering procedures were in accordance with the good animal welfare practices approved by the CHEA rules. The birds were slaughtered, after a fasting time of 16 hours (overnight), by cutting the jugular vein until total bleeding (3 min) to cause the least possible stress to the animal. The carcasses were cooled and maintained at 4°C for 24 hours postmortem. After that, the Pectoralis and Gastrocnemius muscles were withdrawn and stored at −80°C until analysis.

### 2.2. Analytical Determinations

The intramuscular lipids were extracted according to Folch et al. [16]. Briefly, a sample of 4 grams of Pectoralis and Gastrocnemius muscles (free of dissectible visible fat) was homogenized at 35000 rpm with a Virtis 45 during 1 min with 80 ml of chloroform : methanol (2 : 1). Afterward, the homogenate was filtered, moved in a separating funnel, mixed by inversion for one minute, and decanted overnight. The lower phase (chloroform containing lipids) was recuperated in a glass balloon, evaporated at 45°C with a light vacuum in a Rotavapor (IKA basic). The balloon was dried in an oven at 35°C for 60 min and cooled at ambient temperature overnight in a vacuum desiccator. The balloon was weighted to determine the percentage of lipids of each sample. The methylation of fatty acids followed the procedure described by Ichihara et al. [17], using methanolic KOH. The determination of fatty acids by gas chromatography followed the procedure described by Eder [18], using fused-silica capillary column CPSIL-88 of 100 m installed in a split/splitless chromatograph Clarus 500 (Perkin Elmer Instruments, USA). A FID detector and automatic injection of 1 *µ*l of sample with a split of 50% were used. Hydrogen was used as carrier gas. The thermal conditions were injector/detector temperatures 250°C/250°C and oven held at 90°C for one minute after the injection of the sample; after that, the oven temperature was increased to 225°C at 15°C/min. Fatty acids methylated esters (FAMEs) were determined comparing the retention time to authentic standards (Sigma Corp, USA). Individual FAME was quantified as a percentage of total analysed FAMEs.

### 2.3. Calculus of Lipids Indices

The calculus of lipids indices was performed from the data of the fatty acid composition of intramuscular lipids, obtained here. The following indices were calculated.

#### 2.3.1. Index of Atherogenicity (IA)

Compute the relationship between the sum of the main saturated (proatherogenic) and the unsaturated (antiatherogenic) fatty acids. It was calculated according to Ulbricht and Southgate [19] as follows:

(1) $$\begin{array}{c} IA=\frac{\left(4\times C14\text{:}0+C16\text{:}0\right)}{\left[\sum MUFA+\sum \left(n\text{-}6\right)+\sum \left(n\text{-}3\right)\right]}. \end{array}$$

#### 2.3.2. Index of Thrombogenicity (IT)

Estimate the potential to form clots in the blood vessels, determined by the relationship between the prothrombogenic (saturated) and the antithrombogenic fatty acids (sum of MUFA and PUFA). It was calculated according to Ulbricht and Southgate [19] as follows:

(2) $$\begin{array}{c} IT=\frac{\left(C14\text{:}0+C16\text{:}0+C18\text{:}0\right)}{\left[0.5\times \sum MUFA+0.5\times \sum \left(n\text{-}6\right)+3\times \sum \left(n\text{-}3\right)+\sum \left(n\text{-}3\right)/\sum \left(n\text{-}6\right)\right]}. \end{array}$$

#### 2.3.3. Hypocholesterolemic/Hypercholesterolemic Ratio (h/H)

Compute the relation between unsaturated fatty acids (MUFA and PUFA) and the saturated fatty acids 14:0 and 16:0. The h/H ratio was calculated according to Fernández et al. [20], as follows:

(3) $$\begin{array}{c} \frac{h}{H}=\frac{\left(C14\text{:}1+C16\text{:}1+C18\text{:}1+C20\text{:}1+C22\text{:}1+C18\text{:}2+C18\text{:}3+C20\text{:}3+C20\text{:}4+C20\text{:}5+C22\text{:}4+C22\text{:}5+C22\text{:}6\right)}{\left(C14\text{:}0+C16\text{:}0\right)}. \end{array}$$

### 2.4. Enzyme Activity Index

The enzyme activity of desaturase, elongase, and thioreductase was estimated by relating the amount of the specific substrate to the corresponding product of the respective enzyme. The calculated ratios were 16:1n-7 to 16:0 and 18:1n9 to 18:0. The activity of stearoyl-CoA desaturase (delta-9-desaturase) was estimated by calculating the ratio 16:1n-7 + 18:1n-9 to 16:0 + 18:0. The delta-5 desaturase + delta-6 desaturase sum was used as an index for the estimation of the formation of long chain n-6 and n-3 starting from the corresponding precursors C18:2n6 and C18:3n3 [14]. Also, the ratio 18:0 to 16:0 was calculated to estimate the elongase activity. The thioesterase was estimated as the ratio of C16:0 to C14:0 [15].

### 2.5. Statistical Analysis

Data are presented as mean ± SEM. Fatty acid content, lipids indices, and enzyme indexes were analysed by ANOVA with a GLM procedure using diet and muscle type as fixed factors and the interaction diet × muscle (*P* < 0.05). Also, one-way ANOVA was used to determine the effect of the diet for each muscle separately, as well as a Tukey-Kramer post hoc test (*P* < 0.05).

## 3. Results and Discussion

Selenium is an essential micronutrient, which is implicated in various physiological animal functions like growth, fertility, and immunity responses. It plays a crucial role in the defense against the accumulation of hydroperoxides from cellular metabolism [21, 22]. However, it is necessary to consider the toxicity risk of selenium when added in high doses in animal feed [22]. The FDA [23] has approved and advised that the supplementation of selenium in complete feed for chicken, swine, turkey, sheep, cattle, and duck should not exceed a level of 0.3 ppm. The European Food Safety Authority recommended similar levels of selenium supplementation in animal feed [24]. In the present investigation, the organic and the inorganic selenium were added in doses of 0.3 ppm (Table 1). The use of such level ensures the applicability of the results in chicken nutrition, in order to produce meat which is enriched in selenium but contains no risk for consumers.

Nevertheless, another role of selenium has been observed in relation to the modification of the fatty acids composition of chicken meat. Indeed, Haug et al. [21] showed a different repartition of fatty acids, in particular, the n-3 PUFA ones. In the present investigation, the lipids and fatty acids composition of the* Pectoralis* muscle showed a lower content in total lipids than* Gastrocnemius* muscle (Table 2). This is a usual and expected result for chicken [25]. However, the supplementation with selenium did not show any main effect, although there is a significant interaction between muscle and diet. This means that selenium could affect the lipids content depending on the muscle type (Table 2).

The comparison between the fatty acids compositions of the two muscles showed that the* Pectoralis* muscle contained more SFA than the* Gastrocnemius *muscle, principally the C16:0 and the C18:0. When the main effect of diet is considered, it seems that the C14:0 and the C16:0, but not C18:0, showed a reduced level in the two muscles in comparison to the control (Table 2). In the case of C16:0, only Se-Met was effective. This is an interesting finding because it seems that this decreasing effect is specific for those fatty acids, C14:0 and C16:0, which promote the occurrence of cardiovascular diseases in human [26]. This point needs to be considered in future investigations. Concerning MUFA, it seems that the* Gastrocnemius *muscle showed more MUFA than the* Pectoralis* muscle (Table 2) and, as expected, the C18:1 is the most represented fatty acid within the MUFA. Furthermore, the selenium as Se-Met promoted more content of C18:1 when compared to Se-Na and control (Table 2). For PUFA, no significant main effects were observed for muscle or diet (Table 2). However, when the fatty acids were considered individually, it appears that the* Pectoral* showed more PUFA than the* Gastrocnemius *muscle for the most valuable fatty acids for human health such as DPA and DHA. Meanwhile, the two essential PUFA C18:2n6 (linoleic acid) and C18:3n3 (*α*-linolenic acid) showed a higher level in* Gastrocnemius *muscle. It is known that these two fatty acids come exclusively from diet and cannot be synthetized by the tissues. When the main effect of diet is considered, it seems that the C18:3n3 showed a higher level when the selenium, independently of its chemical source, is added in feed in comparison to the control (Table 2). These results are in accord with the findings of Haug et al. [21], Ševčíková et al. [27], and Kralik et al. [28]. Furthermore, a significant interaction has been obtained, suggesting that the selenium could modify the distribution of this fatty acid in chicken meat, depending on the muscles. A similar, but limited, main effect was obtained for C18:2n6. Indeed, only Se-Met increased the level of this fatty acid, in comparison to Se-Na and control (Table 2). The differences in the level of C18:3n3, and on a smaller scale, of C18: 2n6, in comparison to the control, could be explained by the reduced degradation of these fatty acids by the oxidation processes. This protection could be done by the action of glutathione and the enzyme glutathione peroxidase (GPx), through the elimination of free radicals capable of initiating and propagating the fatty acids oxidation, particularly the PUFA ones [29].

Like discussed before, selenium in the chicken diet has been associated in previous investigations with an increase in n-3 fatty acids in* Gastrocnemius *muscle [21, 27] and in* Pectoralis* muscle [28]. Taken together, it was observed, in those investigations, that the increase of the fatty acids level, after supplementation by selenium, happened mainly for the *α*-linolenic acid (*α*-C18:3n3), EPA, DPA, and DHA in* Gastrocnemius *muscle. Unfortunately, in the present investigation, DPA and DHA did not show any significant effect of selenium on their level in comparison to the control. For EPA, the results showed a significant diet main effect when Se-Na is used in comparison to the Se-Met and the control (Table 2). However, the absolute values were very low and incite us to be cautious on the interpretation about the effectiveness of selenium for this fatty acid. Contrarily to our finding and those of Ševčíková et al. [27], Haug et al. [21], and Kralik et al. [28], Zduńczyk et al. did not find any effects of selenium on the fatty acids composition of* Pectoralis* muscle [30]. As it was found in the present study with chicken, the supplementation with selenium affected the fatty composition in other species and products, independently of its chemical form. In ruminant species, the effect of the supplementation with selenium, independently of its form, showed controversial results concerning the modification of lipids content and the fatty acids composition of meat [31]. More investigations should be done to clarify this interesting point linking the supplementation with selenium to the modification of the composition of meat in some specific fatty acids, such as C18:3n3, EPA, and DHA, which have been generally associated with health concerns for human [32].

In the present investigation, the focus on human nutrition and health has been considered through the calculus of some lipids indices associated with the fatty acids composition. These indices are generally used to rank foods in regard to their potential effect on the promotion of cardiovascular diseases. In the present investigation, the PUFA/SFA, n-6, n-3, and the n-6/n-3 ratio yield no different main effects when the two muscles were considered (Table 3). On the contrary, the atherogenic (IA) and thrombogenic (IT) indices showed that the* Pectoralis* muscle had undesirable IA and IT indices when compared to the* Gastrocnemius *muscle. For these two indices, the desirable value has to be as low as possible. The hypocholesterolaemic versus hypercholesteraemic indices (h/H), as opposed to the IA and IT indices, must be as high as possible in order to protect consumer from the hypercholesterolaemia, a factor which promotes the atherosclerosis syndrome in humans [33]. The h/H obtained in the present investigation had a better value in the* Pectoralis *muscle than in the* Gastrocnemius *muscle (Table 3). However, there is no effect of the supplementation of selenium, in its two forms, on the health scope. For all these considered indices, no main effects have been observed concerning selenium, independently of its form (Table 3). Nonetheless, there is an interaction (muscle × diet) for the n-3 fatty acids, the n-6/n-3 ratio, and the h/H indices. These last results encourage us to carry on, in order to have a better understanding of the action of selenium in the repartition of fatty acids in chicken meat.

Finally, one of the goals of the present investigation was to compare the effect of feed supplementation with organic and inorganic selenium on the activity of desaturase, elongase, and thioesterase enzymes in the two muscles, through the calculus of a few indexes related to the activity of those enzymes. The enzyme activities were estimated by relating the amount of the specific substrate to the corresponding product of the respective enzyme. These indexes can be used as surrogates of the measure of the true enzyme activities [34]. The delta-9-desaturase can be estimated by the ratio C16:1/C16:0 specifically for the C16:1 and by the ratio C18:1/C18:0. This last one is specific for the desaturation of C18:1, the main MUFA present in chicken meat [27]. Meanwhile, the total delta-9 desaturase index (for both C16:1 and C18:1) can be estimated by the sum of the previous two indexes [14, 35]. Furthermore, there is a convenient way to estimate the total activities of delta-5-desaturase and delta-6-desaturase, by using a similar approach. These two desaturases are the key enzymes catalysing the formation of n-6 and n-3 PUFA starting from their precursors C18:2n6 and C18:3n3 [14]. The elongase and the thioesterase enzymes can be estimated by the same procedure based on the calculus of the ratios C18:0/C16:0 and C16:0/C14:0, respectively. The thioesterase is responsible for terminating the fatty acids synthesis and release of the neosynthetized fatty acids, mainly C16:0 and C14:0. In the current investigation, a main muscle effect has been obtained only for the desaturase enzymes, showing that* Gastrocnemius *muscle has more desaturases activities than the* Pectoralis* muscle (Table 4). This observation could explain why the* Gastrocnemius* muscle contains more MUFA, such as C14:1, C16:1, and C18:1 (Table 2), which is probably due to the action of delta-9-desaturase. Likewise, more C18:2n6 and C18:3n3 could be due to the action of the delta-5 and delta-6 desaturase (Table 2). When the effect of selenium is analysed, only the index for thioesterase showed a significant main effect. The results showed that the Se-Na induced a higher index in comparison to the control (Table 4). Note that a higher index for thioesterase means a lower release of de novo synthetized C14:0 and C16:0. Perhaps this result explains why there is a significant main effect due to the selenium, since meat has a lower level of those two atherogenics fatty acids. The two forms of selenium and Se-Met reduced significantly the level of C14:0 and C16:0, respectively (Table 2).

## 4. Conclusions

It seems that selenium, independently of its chemical form, reduced the level of the two most atherogenic fatty acids, C14:0 and C16:0, present in chicken meat. At the same time, the Se-Met raised the beneficial polyunsaturated fatty acids, such as the C18:2n6 and particularly the C18:3n3. This interesting and favourable unbalanced repartition between the atherogenic fatty acids and the beneficial PUFA, caused by the selenium in chicken meat, seems to be promising and should be carefully considered in future investigations. Furthermore, even though the effect of selenium on the enzymes implicated in the fatty acid synthesis, desaturation, and elongation remains inconclusive in the present investigation, it seems it would be of great interest to include it in future studies. Selenium, and particularly Se-Met, could be used, within approved doses, as tools to modify the profile of fatty acids in chicken meat, in order to move towards a more convenient composition of fatty acids regarding the human health.

## Figures and Tables

**Table 1:** Chemical composition of experimental diets.

Items (%)	Experimental diets		
	Control	Se-Met (0.30 ppm Se)	Se-Na (0.30 ppm Se)
Ground corn	60.0	60.0	60.0
Soybean meal	32.5	32.5	32.5
Meat and bone meal	4.20	4.20	4.20
Calcium carbonate	1.00	1.00	1.00
Dicalcium phosphate	0.80	0.80	0.80
Sunflower oil	0.50	0.50	0.50
Salt	0.30	0.30	0.30
Lysine	0.25	0.25	0.25
Dl-Methionine	0.10	0.10	0.10
Premix^*∗*^	0.25	0.25	0.25

*Chemical composition*			
Crude protein, (%)^*∗∗*^	20.0	20.0	20.0
ME, (MJ/kg)	2931	2931	2931
Crude fiber, (%)^*∗∗*^	3.70	3.70	3.70
Ca, (%)^*∗∗*^	1.20	1.20	1.20
P, (%)^*∗∗*^	0.42	0.42	0.42
Se, (ppm)^*∗∗*^	0.01	0.29	0.30

^*∗*^Mineral and vitamin premix. Provided (per 1 kilogram): 8.000.000 IU of vitamin A, 1.300.000 IU of vitamin D3, 16.000 IU of vitamin E, 5 g of vitamin K, 3.5 g of vitamin B2, 6.5 g of calcium D-pantothenate, 20 g of niacin, 0.3 g of folic acid, 8.5 mg of vitamin B12, 350 g of choline chloride, 0.3 g of vitamin B1, 0.6 g of vitamin B6, 60 g of Mn, 25 g of Zn, 16 g of Fe, 1 g of Cu, 1 g of I, and 60 mg of Co. ME: metabolizable energy. ^*∗∗*^Analyzed values.

**Table 2:** Fatty acids composition (g/100 g of total fatty acids) of *Pectoralis* and *Gastrocnemius* muscles of chickens supplemented in diet with inorganic (Se-Na) or organic (Se-Met) selenium.

	*Pectoralis* (Pe)			*Gastrocnemius* (G)			Main effects		
	Control	Se-Na	Se-Met	Control	Se-Na	Se-Met	Muscle (M)	Diet (D)	M × D
Lipids %	1.77 ± 0.11	1.41 ± 0.17	1.43 ± 0.05	2.44 ± 0.31	3.01 ± 0.27	3.09 ± 0.24	*P* < 0.01 G > Pe	NS	*P* < 0.04
Fatty acids %									
C14:0	0.69 ± 0.10	0.60 ± 0.17	0.62 ± 0.08	0.73 ± 0.04	0.61 ± 0.18	0.61 ± 0.13	NS	*P* < 0.03 Se < C	NS
C14:1	0.12 ± 0.03	0.10 ± 0.01	0.09 ± 0.01	0.17 ± 0.03	0.12 ± 0.01	0.11 ± 0.02	*P* < 0.01 G > Pe	*P* < 0.01 Se < C	NS
C16:0	26.6 ± 3.20	27.0 ± 2.54	26.6 ± 1.61	26.8 ± 0.62	25.6 ± 2.91	24.0 ± 1.60	*P* < 0.04 Pe > G	*P* < 0.05 Se-Met < C	NS
C16:1	4.21 ± 1.92	4.22 ± 0.85	3.78 ± 0.21	5.58 ± 1.51	5.54 ± 1.08	4.52 ± 0.62	*P* < 0.01 G > Pe	*P* < 0.05 Se < C	NS
C18:0	10.2 ± 2.29	9.99 ± 0.72	9.82 ± 0.47	8.65 ± 1.93	8.80 ± 1.34	9.18 ± 1.04	*P* < 0.01 Pe > G	NS	NS
C18:1	33.6 ± 2.10	34.5 ± 1.73	35.1 ± 2.69	35.2 ± 3.21	37.5 ± 2.19	37.1 ± 3.27	*P* < 0.01 G > Pe	*P* < 0.03 Se-Met > C	NS
C18:2n6	15.7 ± 1.92	14.4 ± 1.10	16.0 ± 2.43	16.7 ± 1.26	15.8 ± 1.15	17.5 ± 2.37	*P* < 0.01 G > Pe	*P* < 0.03 Se-Met > Se-Na	NS
C20:1	0.18 ± 0.09	0.20 ± 0.08	0.16 ± 0.02	0.18 ± 0.02	0.20 ± 0.10	0.33 ± 0.13	*P* < 0.02 G > Pe	*P* < 0.05 Se-Met > C	*P* < 0.01
C18:3n3	0.35 ± 0.06	0.46 ± 0.09	0.43 ± 0.12	0.44 ± 0.02	0.42 ± 0.02	0.81 ± 0.11	*P* < 0.01 G > Pe	*P* < 0.01 Se > C	*P* < 0.01
C20:3n6	0.27 ± 0.14	0.22 ± 0.08	0.20 ± 0.10	0.15 ± 0.05	0.15 ± 0.06	0.21 ± 0.06	*P* < 0.01 Pe > G	NS	NS
C20:3n3	0.23 ± 0.12	0.30 ± 0.09	0.20 ± 0.08	0.19 ± 0.10	0.22 ± 0.02	0.16 ± 0.05	*P* < 0.01 Pe > G	*P* < 0.01 Se-Met < Se-Na	NS
C22:1	0.64 ± 0.31	0.65 ± 0.17	0.60 ± 0.13	0.46 ± 0.28	0.40 ± 0.11	0.35 ± 0.03	*P* < 0.01 Pe > G	NS	NS
C20:4n6 ARA	3.47 ± 1.65	3.36 ± 0.82	3.23 ± 0.83	2.17 ± 0.97	2.19 ± 0.63	2.25 ± 0.35	*P* < 0.01 Pe > G	NS	NS
C22:4n6	1.03 ± 0.61	0.96 ± 0.32	0.81 ± 0.35	0.62 ± 0.30	0.56 ± 0.20	0.56 ± 0.07	*P* < 0.01 Pe > G	NS	NS
C20:5n3 EPA	0.05 ± 0.02	0.08 ± 0.04	0.04 ± 0.01	0.07 ± 0.07	0.05 ± 0.02	0.03 ± 0.01	NS	*P* < 0.03 Se-Met < Se-Na	*P* < 0.03
C22:5n3 DPA	0.35 ± 0.17	0.26 ± 0.08	0.26 ± 0.12	0.18 ± 0.11	0.17 ± 0.06	0.19 ± 0.04	*P* < 0.01 Pe > G	NS	NS
C22:6n3 DHA	0.26 ± 0.13	0.23 ± 0.09	0.20 ± 0.08	0.13 ± 0.02	0.14 ± 0.06	0.13 ± 0.03	*P* < 0.01 Pe > G	NS	NS
Unidentified	2.10 ± 0.20	2.45 ± 1.18	1.87 ± 0.82	1.52 ± 0.48	1.50 ± 0.25	2.01 ± 0.75	—	—	—
SFA	37.5 ± 1.07	37.6 ± 0.99	37.0 ± 0.74	36.2 ± 0.54	35.0 ± 2.10	33.8 ± 0.80	*P* < 0.01 Pe > G	NS	NS
MUFA	38.7 ± 3.15	39.6 ± 1.96	39.8 ± 2.71	41.6 ± 4.51	43.8 ± 3.20	42.4 ± 3.79	*P* < 0.01 G > Pe	NS	NS
PUFA	21.1 ± 1.87	19.7 ± 1.23	20.8 ± 1.45	20.3 ± 1.16	19.4 ± 0.82	21.5 ± 1.34	NS	NS	NS

Data are mean ± SEM. NS: not significant, C: control, Se: effect of selenium from Se-Na and Se-Met, SFA: saturated fatty acids, MUFA: monounsaturated fatty acids, and PUFA: polyunsaturated fatty acids.

**Table 3:** Calculated lipids indices in terms of the health needs of consumers for *Pectoralis* and *Gastrocnemius* muscles of chickens supplemented in diet with inorganic (Se-Na) or organic (Se-Met) selenium.

	*Pectoralis* (Pe)			*Gastrocnemius* (G)			Main effects		
	Control	Se-Na	Se-Met	Control	Se-Na	Se-Met	Muscle (M)	Diet (D)	M × D
P/S	0.58 ± 0.03	0.54 ± 0.02	0.58 ± 0.05	0.57 ± 0.02	0.58 ± 0.05	0.65 ± 0.04	NS	NS	NS
n-6	20.5 ± 3.31	19.0 ± 2.17	20.2 ± 2.68	19.6 ± 2.57	18.7 ± 1.67	20.5 ± 2.61	NS	NS	NS
n-3	1.23 ± 0.22	1.33 ± 0.19	1.13 ± 0.11	1.02 ± 0.12	0.98 ± 0.07	1.32 ± 0.07	NS	NS	*P* < 0.02
n-6/n-3	18.2 ± 2.82	14.9 ± 1.60	18.0 ± 0.79	19.7 ± 1.24	19.7 ± 2.33	15.6 ± 0.96	NS	NS	*P* < 0.01
IA	0.49 ± 0.01	0.49 ± 0.01	0.48 ± 0.02	0.48 ± 0.02	0.45 ± 0.01	0.41 ± 0.01	*P* < 0.01 Pe > G	NS	NS
IT	1.14 ± 0.05	1.15 ± 0.05	1.13 ± 0.04	1.09 ± 0.02	1.04 ± 0.09	0.96 ± 0.03	*P* < 0.03 Pe > G	NS	NS
h/H	2.23 ± 0.014	2.25 ± 0.02	2.17 ± 0.12	2.45 ± 0.21	2.23 ± 0.09	2.61 ± 0.12	*P* < 0.01 G > Pe	NS	*P* < 0.04

Data are mean ± SEM. NS: not significant, P/S: PUFA/SFA, IA: atherogenicity, IT: thrombogenicity, and h/H: hypocholesterolemic/hypercholesterolemic ratio.

**Table 4:** Enzymes indexes of fatty acid metabolism estimated on the basis of fatty acid composition of *Pectoralis* and *Gastrocnemius* muscles of chickens supplemented in diet with inorganic (Se-Na) or organic (Se-Met) selenium.

Enzyme indexes	*Pectoralis* muscle (Pe)			*Gastrocnemius* muscle (G)			Main effects		
	Control	Se-Na	Se-Met	Control	Se-Na	Se-Met	Muscle (M)	Diet (D)	M × D
Δ-9 desaturase									
16:1/16:0	0.15 ± 0.01	0.16 ± 0.01	0.14 ± 0.01	0.21 ± 0.01	0.22 ± 0.02	0.19 ± 0.01	*P* < 0.001 G > Pe	NS	NS
18:1/18:0	3.46 ± 0.34	3.47 ± 0.11	3.59 ± 0.13	4.22 ± 0.37	4.38 ± 0.33	4.11 ± 0.27	*P* < 0.002 G > Pe	NS	NS
16:1 + 18:1/16:0 + 18:0	1.03 ± 0.03	1.04 ± 0.02	1.07 ± 0.03	1.15 ± 0.05	1.27 ± 0.07	1.26 ± 0.05	*P* < 0.001 G > Pe	NS	NS
Δ-5 + Δ-6 desaturases	19.8 ± 2.62	20.6 ± 1.05	18.7 ± 1.60	12.7 ± 1.16	13.5 ± 0.94	12.5 ± 0.55	*P* < 0.001 G < Pe	NS	NS
Elongase 18:0/16:0	0.39 ± 0.04	0.37 ± 0.02	0.37 ± 0.01	0.32 ± 0.02	0.34 ± 0.01	0.39 ± 0.02	NS	NS	NS
Thioesterase 16:0/14:0	38.8 ± 0.68	46.5 ± 2.55	43.3 ± 1.37	36.6 ± 0.42	43.4 ± 2.59	40.0 ± 2.01	NS	*P* < 0.001	NS
							Se-Na > Control		

Results are means ± SEM. Δ-9 desaturase indexes, Δ-5 + Δ-6 desaturases, elongase, and thioesterase indexes were calculated according to [14, 15].
